# P-721. Estimated Costs of Worldwide Vaccination for HPV

**DOI:** 10.1093/ofid/ofaf695.932

**Published:** 2026-01-11

**Authors:** Gabriel Samanta, Namwa Wongkalasin, Andrew Hill, Cassandra Fairhead

**Affiliations:** Imperial College London, London, England, United Kingdom; Imperial College London, London, England, United Kingdom; University of Liverpool, London, England, United Kingdom; Royal Free Hospital, London, London, England, United Kingdom

## Abstract

**Background:**

In 2022 there were over 925,000 cases of human papillomavirus (HPV)-related cancer, with over 75% occurring in low- and middle-income countries (LMICs). 350,000 women die from cervical cancer annually. Highly effective HPV vaccines are available, with striking public health impact when uptake is high. The WHO aims for 90% coverage for women and girls worldwide, but coverage remains low (20% for adolescent girls, 7% for boys). Multiple barriers exist to reaching this target, with a key factor being cost. There is a gross variation between HPV vaccine prices, from $323 (nonvalent) to $2.44 (generic quadrivalent), per dose. The patent for Gardasil is expected to expire around 2028. We conducted an economic analysis using projections of different vaccine programme scenarios to show the impact of pricing mechanisms on vaccine coverage.

**Methods:**

We compared the cost of immunising with Gardasil-4, Gardasil-9, and generic alternatives using data from the WHO Vaccine Price dataset (2021-2023), UNICEF, and PAHO. Single-age cohort data for the estimated number of 9-year-olds were obtained from World Bank estimates. HPV vaccine use by country was collected from the WHO coverage dataset.

The maximum purchasable number of doses of each of the three vaccines was calculated using respective vaccine costs and specific country budgets. Total costs for achieving 90% coverage were calculated using population and vaccine price data.

Finally, we analysed a comparator scenario where countries included in the Medicine Patent Pool (MPP) Cabotegravir voluntary licenses could access HPV vaccines at consistently low prices for 124 LMICs, versus tiered pricing through GAVI.

**Results:**

Switching to generic alternatives and single-age, single dose programmes could save up to $8.37 billion per year. (Figure 1).

In Cabotegravir-licensed countries, vaccinating 90% of children with branded vaccine costs surpass current health budgets. Generics could allow for coverage exceeding this target by 3.1 times.

**Conclusion:**

Worldwide vaccination of all 9-year-olds using single-dose generic HPV vaccines could cost $302M. This is 97% lower than the worldwide HPV vaccine sales of $8.7 billion. Expanding mechanisms such as MPP-coordinated licencing to HPV vaccination could increase HPV vaccine access, particularly in LMICs.

**Disclosures:**

All Authors: No reported disclosures

**Figure 1:**
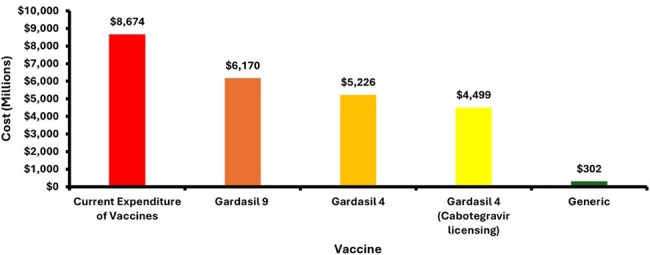
Global cost of vaccines to vaccinate 90% of girls and boys aged 9 years old against HPV with different vaccine options.

**Figure 2:**
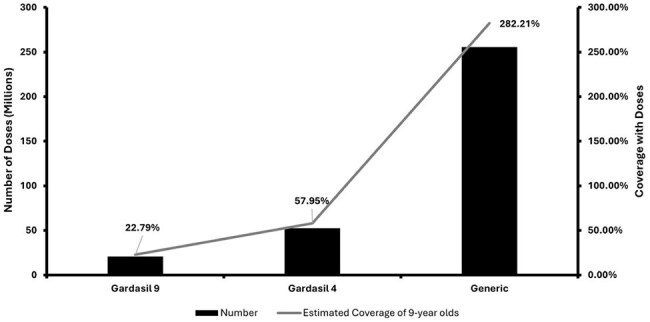
Number and coverage (of 9-year-olds) of vaccines purchasable with current expenditure in Cabotegravir Licensed countries

